# Validation of Biomarkers for Distinguishing *Mycobacterium tuberculosis* from Non-Tuberculous Mycobacteria Using Gas Chromatography−Mass Spectrometry and Chemometrics

**DOI:** 10.1371/journal.pone.0076263

**Published:** 2013-10-17

**Authors:** Ngoc A. Dang, Sjoukje Kuijper, Elisabetta Walters, Mareli Claassens, Dick van Soolingen, Gabriel Vivo-Truyols, Hans-Gerd Janssen, Arend H. J. Kolk

**Affiliations:** 1 Analytical Chemistry and Forensic Science, University of Amsterdam, Amsterdam, The Netherlands; 2 Desmond Tutu TB Centre, Department of Paediatrics and Child Health, Stellenbosch University, Tygerberg, South Africa; 3 National Institute of Public Health and the Environment (RIVM), Bilthoven, The Netherlands; 4 Department of Clinical Microbiology and Department of Pulmonary Diseases, Radboud University, Nijmegen Medical Centre, Nijmegen, The Netherlands; 5 Unilever Research and Development, Vlaardingen, The Netherlands; BarcelonaUniversity Hospital, Spain

## Abstract

Tuberculosis (TB) remains a major international health problem. Rapid differentiation of *Mycobacterium tuberculosis* complex (MTB) from non-tuberculous mycobacteria (NTM) is critical for decisions regarding patient management and choice of therapeutic regimen. Recently we developed a 20-compound model to distinguish between MTB and NTM. It is based on thermally assisted hydrolysis and methylation gas chromatography-mass spectrometry and partial least square discriminant analysis. Here we report the validation of this model with two independent sample sets, one consisting of 39 MTB and 17 NTM isolates from the Netherlands, the other comprising 103 isolates (91 MTB and 12 NTM) from Stellenbosch, Cape Town, South Africa. All the MTB strains in the 56 Dutch samples were correctly identified and the model had a sensitivity of 100% and a specificity of 94%. For the South African samples the model had a sensitivity of 88% and specificity of 100%. Based on our model, we have developed a new decision-tree that allows the differentiation of MTB from NTM with 100% accuracy. Encouraged by these findings we will proceed with the development of a simple, rapid, affordable, high-throughput test to identify MTB directly in sputum.

## Introduction

Tuberculosis (TB) remains a major international health threat, with 8.7 million new cases and 1.4 million deaths in 2011 [1]. The global emergence of both human immunodeficiency virus (HIV) infection and multidrug resistant TB (MDR-TB) poses significant threats to TB control. An estimated 13% of new TB cases occur in those infected with HIV [1]. Up to 10% of people with latent TB will develop active disease [2], but HIV co-infection might increase this risk almost 40 fold [3]–[5]. Several methods are available for the diagnosis of TB, but all have limitations [6]–[8]. Worldwide, direct identification of mycobacteria in sputum using Ziehl-Neelsen staining and microscopy is still the most commonly used method. However, the sensitivity of the test varies considerably between 30 and 70% [8]. Furthermore the Ziehl-Neelsen test cannot distinguish *Mycobacterium tuberculosis* complex (MTB) from non-tuberculous mycobacteria (NTM). Rapid culture systems have been developed, for example, the Mycobacteria Growth Indicator Tube (MGIT) method [9] and the Microscopic Observation Drug Susceptibility assay [10]. Again these tests do not differentiate between MTB and NTM. This distinction, however, is essential to ensure the correct choice of therapy.

The diagnosis of TB is more complicated in HIV-positive persons because of a higher frequency of negative and paucibacillary sputum smears (i.e negative Ziehl-Neelsen test) [11]. In a study in Khayelitsha, a district in Cape Town, South Africa, 49% of HIV-positive patients on TB treatment had a negative smear although the sputum culture was positive [12]. Thus time-consuming culture is still necessary to confirm a diagnosis of TB in HIV positive patients [13]. Since it can take up to three weeks to obtain results from culture using the traditional phenotypic diagnostic techniques to distinguish MTB from NTM [6], there is a great need for a rapid, affordable and sensitive method for the early diagnosis of TB that will then allow appropriate and effective therapy.

NTM are an increasing problem, particularly for those with HIV or chronic lung disease [14], [15]. In patients with suspected tuberculosis in Cape Town, South Africa, NTM rather than MTB were grown from approximately 10% of the culture-positive but smear-negative sputum samples [16]. Modern nucleic acid amplification techniques rapidly distinguish MTB from NTM, but these techniques are not widely available in resource-limited settings due to the high cost and lack of infrastructure and expertise. Diagnostic delay contributes to ongoing transmission and poor clinical outcomes. There are commercially available gas chromatography techniques for distinguishing different types of mycobacteria. However, these do not lend themselves to further development for use in resource-constrained countries.

Recently we described a new approach for the identification of biomarkers to differentiate MTB from NTM in early cultures. We used thermally-assisted hydrolysis and methylation followed by gas chromatography-mass spectrometry (THM-GC-MS) and chemometrics [17]. Our aim is to develop an affordable and practical test using these biomarkers for the direct identification of mycobacteria in sputum and to apply this technology in a portable device. This would allow the rapid classification of patients with suspected TB into three categories: those with MTB, those with NTM and those with no mycobacteria in their sputum. Our model identified 20 compounds that could distinguish 15 MTB from 29 NTM cultivated strains with 95% accuracy. To further test the model we describe here its application to 56 well-characterized mycobacterial isolates from patients in the Netherlands and 103 primary isolates from patients from Stellenbosch, Cape Town, South Africa.

## Materials and Methods

### Culture of mycobacteria

Fifty-six mycobacterial strains were obtained from patients in the Netherlands, and identified at the National Institute for Public Health and the Environment (RIVM) in the Netherlands (Table 1). These constitute Testset-1. In the Netherlands all hospitals are required by law to send all mycobacterial isolates, whether MTB or NTM, to the Dutch Mycobacteria Reference Laboratory at the RIVM for species determination, drug susceptibility testing and strain typing for contact investigations. The 56 strains were selected by the RIVM to provide representative examples of the MTB and NTM strains found in the Netherlands. The strains were cultured using the Mycobacteria Growth Indicator Tube (MGIT) culture system (MGIT, BD Diagnostics, Detroit, MI, USA). The species was determined using the line probe assay (GenoType Mycobacterium, Hain Life Science GmbH, Nehren, Germany). A separate set, Testset-2, consisting of 103 mycobacterial isolates was obtained from patients with suspected pulmonary TB in Stellenbosch, Cape Town, South Africa (Table 2). These strains were cultured from sputum samples using the manual BACTEC MGIT reader. A positive MGIT result was confirmed by the Bioline test SD TB Ag MPT64 (Standard Diagnostics Inc, Kyonggi-do, South Korea) to discriminate between MTB and NTM and the species was subsequently determined by 16S ribosomal RNA sequencing (3730XL Genetic Analyzer, Applied Biosystems, Carlsbad, CA, USA). The MGIT tubes were labelled with a code and later shipped to Amsterdam on dry ice.

**Table 1:** The 56 mycobacterial strains from The Netherlands obtained via The National Institute for Public Health and the Environment (RIVM).

33 strains *M. tuberculosis*	2 strains *M. gordonae*
2 strains *M. africanum*	2 strains *M. kansasii type I*
2 strains *M. bovis spp bovis*	2 strains *M. malmoense*
2 strains *M. bovis BCG*	1 strain *M. abscessus*
2 strains *M. avium complex*	1 strain *M. haemophilum*
2 strains *M. chelonae complex*	1 strain *M. simiae*
2 strains *M. fortuitum complex*	1 strain *M. marinum*
1 strain *M. intracellulare*	

**Table 2:** 103 primary isolates from Stellenbosch, Cape Town, South Africa.

91 strains *M. tuberculosis*	2 strains *M. avium*
2 strains *M. intracellulare*	1 strain *M. lentiflavum*
2 unknown NTM strains Bioline test Negative	2 strains *M. gordonae*
2 strains *M. peregrinum/M. septicum*	1 strain *N. shimofusensis*

### Sample preparation for thermochemolysis GC-MS

All samples were tested blindly without foreknowledge of whether they contained MTB or NTM. The pellets in the positive MGIT tubes were collected by a sterile Pasteur pipette, and transferred to a 2 mL screw cap vial. The mycobacteria were killed by heating for 20 min at 80°C and the tube was centrifuged at 12,000×g for 10 min. The bacterial pellets were washed with deionized water under the same conditions. The washed pellets were resuspended in deionized water to a concentration of approximately 6×10^8^ bacteria/mL (Mc Farland turbidity 2). Fifteen microliters of the sample was used for the THM-GC-MS analysis.

### Reagents

A 25% tetramethyl ammonium hydroxide (TMAH) solution in methanol was obtained from Sigma–Aldrich (Zwijndrecht, The Netherlands). Before use the solution was diluted ten times with deionized water obtained from a Sartorius Arium 611 UV water purification device (Sartorius, Nieuwegein, The Netherlands). The solution was stable for two weeks at room temperature.

### Instrumentation

All THM–GC–MS experiments were carried out on a Shimadzu GCMS-QP2010 (Shimadzu, Den Bosch, The Netherlands). The GC system was equipped with a “Focus” XYZ robotic auto sampler and an Optic 3 Programmed Temperature Vaporizing (PTV) injector (ATAS GL, Eindhoven, The Netherlands).

### Automated THM-GC-MS procedure

The automated THM-GC-MS procedure has been described previously [18]. In brief, 15 µL of each mycobacterial suspension was first injected into the PTV injector at 40°C. The injector was then rapidly heated to 120°C to eliminate water while retaining the sample in the sintered-bed liner inside the injector. After cooling the injector to 40°C, 20 µL of the 2.5% TMAH reagent was injected to cover the whole bacterial sample. Subsequently, the injector was heated to 120°C to remove the solvent and incubate the residue present in the sintered-bed of the liner. The injector temperature was then increased to 450°C to perform thermochemolysis. After 5 min the injector temperature was decreased and maintained at 320°C until the end of the GC run. All GC analyses were performed on a TC 5MS column (GL Sciences, Tokyo, Japan) of 30 m×0.25 mm internal diameter, coated with 0.25 µm of a 5% phenyl-methylpolysiloxane stationary phase. Helium was used as the carrier gas. The separation was performed by starting the GC oven at 40°C for 3 min, followed by a first ramp of 20°C/min to 100°C with a hold of 7 min, and then a second ramp of 5°C/min to 320°C with a final hold of 6 min. The MS was operated in the full scan mode collecting spectra at a rate of 5 Hz over the mass window from 60 to 500 amu. All samples were randomly and blindly tested.

### Chemometric method

The partial least square discriminant analysis (PLS-DA) model we developed to classify samples as NTM or MTB is based on the (relative) concentration levels of 20 compounds in the THM-GC-MS chromatograms [17]. Briefly; to classify an unknown sample, a THM-GC-MS chromatogram is recorded for the sample and the peak areas of the 20 target compounds are integrated at specific mass channels. Then the areas are normalized to give a total sum of 1 and the following equation is applied: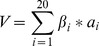, where 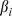 are the coefficients provided in Table 3 and 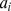 are the normalized areas for the 20 compounds as measured from the target m/z fragment. The value of *V* is then compared with the threshold value. The threshold value is determined by the so-called cost function, i.e. by the importance of a false-positive versus a false-negative classification. The user determines the value for the false-positive rate (here defined as the percentage of non-tuberculosis samples that will be wrongly classified as *M. tuberculosis*) in the Receiver Operating Characteristic (ROC) curve [17]. The ROC curve describes the relationship between the false-positive rate and the true-positive rate (the percentage of *M. tuberculosis* samples correctly classified as MTB). Therefore, a decision on the acceptable false-positive rate implicitly establishes a value for the true-positive rate. Once the false-positive rate (or the true-positive rate) has been set, a threshold value is obtained. A value of *V* below the threshold indicates the presence of an MTB, whereas a value of *V* above the threshold indicates the presence of an NTM. Hence, compounds with negative
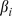
values tend to be dominant in the MTB complex group; while positive
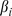
values indicate the compounds have a higher probability of being found in the NTM group. For cases where the sensitivity (true-positive rate) and specificity (true-negative rate) are equally important, the threshold value for *V* is 0.55. In this case, both sensitivity and specificity are 95%.

**Table 3:** Compounds identified as relevant for the differentiation of NTM and MTB strains.

No.	Retention time (min)	Name of compounds	FAMEs^1^	m/z	Beta-coefficients
1	25.01	Methyl tetradecanoate (C14)	C15H30O2	74	3.305
2	29.07	9-Hexadecenoic acid, methyl ester	C17H32O2	83	0.863
3	29.48	Hexadecanoic acid, methyl ester (C16)	C17H34O2	87	1.634
4	30.75	1-Nonadecene	C19H38	97	1.278
5	31.40	Heptadecanoic acid, methyl ester (C17)	C18H36O2	74	−2.819
6	32.75	9-Octadecenoic acid (Z)-, methyl ester	C19H36O2	69	1.153
7	33.33	Octadecanoic acid, methyl ester (C18)	C19H38O2	298	−0.712
8	34.02	Octadecanoic acid, 10-methyl-, methyl ester(TBSA)	C20H40O2	312	0.034
9	36.50	alpha-D-Glucopyranoside, 2,3,4,6-tetra-O-methyl- alpha-D-glucopyranosyl 2,3,4,6-tetra-O-methyl	C20H38O11	71	−1.112
10	40.17	Docosanoic acid, methyl ester (C22)	C23H46O2	354	0.942
11	43.22	Tetracosanoic acid, methyl ester (C24)	C25H50O2	382	3.563
12	43.94	Unknown fatty acid	–	88	−0.580
13	44.09	Tetracosanoic acid, 2,4,6-trimethyl-, methyl ester (C27)	C28H56O2	101	2.966
14	44.23	Tetracosanoic acid, 2,4,6,8-tetramethyl-, methyl ester (C28)	C29H58O2	101	2.961
15	44.70	Pentacosanoic acid, methyl ester (C25)	C26H52O2	87	−0.965
16	46.12	Hexacosanoic acid, methyl ester (C26)	C27H54O2	410	−6.948
17	46.88	Hexacosanoic acid, 2,4,6-trimethyl-, methyl ester (C29)	C30H60O2	101	−2.025
18	47.01	Hexacosanoic acid, 2,4,6,8-tetramethyl-, methyl ester (C30)	C31H62O2	101	−1.935
19	49.55	Octacosanoic acid, 2,4,6,8-tetramethyl-, methyl ester (A)2 (C32)	C33H66O2	101	−0.899
20	49.66	Octacosanoic acid, 2,4,6,8-tetramethyl-, methyl ester (B)2 (C32)	C33H66O2	101	−0.705

1 FAMEs  =  Fatty Acid Methyl Esters.

2 A and ^2^B C32 mycocerosate  =  Two isomers of C32 mycocerosate.

### Ethical approval

This study was approved by the Health Research Ethics Committee at Stellenbosch University, South Africa (reference number N06/09/186).

## Results and Discussion

**Figure 1:**
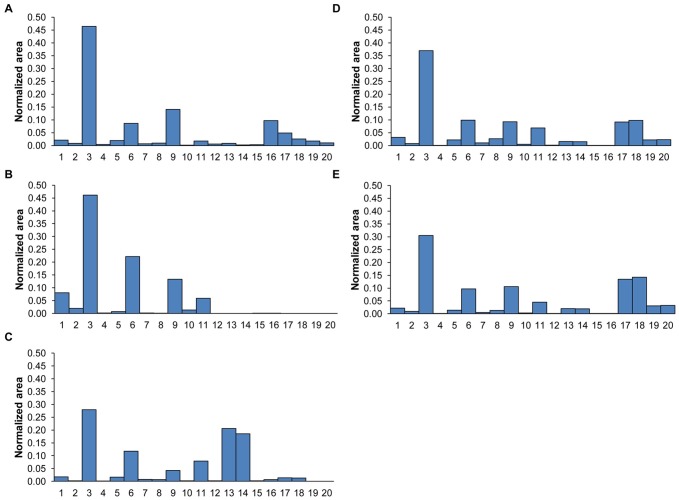
Fingerprint patterns of the normalized areas of the 20 markers in samples from the Netherlands. The graph shows the fingerprint patterns of the normalized areas of 20 marker compounds in different mycobacteria from the Netherlands with (A) *M. tuberculosis*, (B) *M. gordonae*, (C) *M. marinum,* and (D) and (E) two *M. kansasii* type I strains. Case (A) belongs to the MTB group; cases (B–E) belong to the NTM group. Compounds 1–20 are identified in Table 3.

The 56 samples from the Netherlands (Testset-1) consisted of 39 MTB complex strains and 17 opportunistic NTM strains (Table 1). The demographics and clinical characteristics of the population of origin for these samples were not collected, but the strains were representative of those found in the Netherlands. The 56 strains were tested blindly using our THM-GC-MS method. The results using the 20-compound model at the threshold value of 0.55 are summarized in Table 4. Fingerprint patterns of the normalized areas of the 20 compounds for some representative strains are given in Figure 1.

**Table 4:** Results of the analysis of 56 mycobacterial strains from patients in the Netherlands using THM-GC-MS and the 20-compound model.

Mycobacterial species/strain	Classification by THM-GC-MS^1^ using the 20- compound model
33 strains *M. tuberculosis*	33 MTB^2^ complex
2 strains *M. africanum*	2 MTB complex
2 strains *M. bovis spp bovis*	2 MTB complex
2 strains *M. bovis BCG*	2 MTB complex
2 strains *M. avium complex*	2 NTM^3^
2 strains *M. chelonae complex*	2 NTM
2 strains *M. fortuitum complex*	2 NTM
2 strains *M. gordonae*	2 NTM
2 strains *M. malmoense*	2 NTM
1 strain *M. kansasii type I*	1 NTM
1 strain *M. kansasii type I*	1 MTB complex
1 strain *M. abscessus*	1 NTM
1 strain *M. haemophilum*	1 NTM
1 strain *M. marinum*	1 NTM
1 strain *M. simiae*	1 NTM
1 strain *M. intracellulare*	1 NTM

1 THM-GC-MS  =  Thermally-assisted hydrolysis and methylation gas chromatography-mass spectrometry.

2 MTB complex  =  *M. tuberculosis* complex.

3 NTM  =  Non-tuberculous mycobacteria.

**Figure 2:**
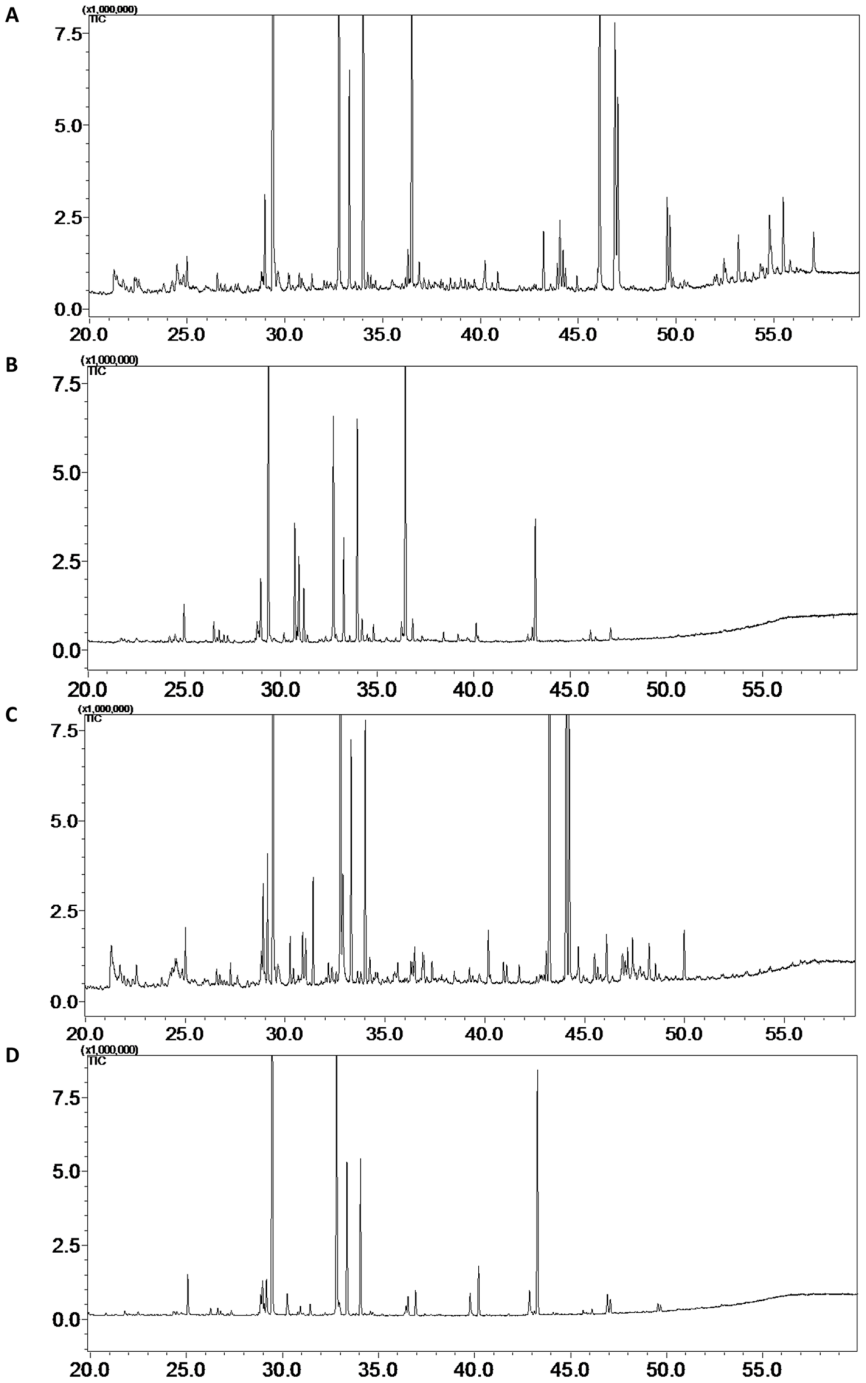
Representative examples of the THM-GC-MS chromatograms. Chromatograms are shown for A: *M. tuberculosis,* B: *M. avium,* C: *M. marinum*, D: *M. kansasii*.

The THM-GC-MS patterns of MTB strains differed notably from those of most NTM strains. We have shown in Figure 2 representative examples of the THM-GC-MS chromatograms for A: *M. tuberculosis,* B: *M. avium,* C: *M. marinum,* D: *M. kansasii.* Clearly MTB strains contained the mycocerosate markers (compounds 17–20 in Table 3). The mycoserates, breakdown products of phthiocerol dimycocerosates (PDIMs), were the most useful for distinguishing between NTM and MTB, since most NTM lack these markers. However, we identified a group of opportunistic NTM mycobacteria e.g. *M. kansasii* that have a pattern of mycocerosate markers very similar to that of the MTB complex. The identification of these NTM strains posed a challenge to our model. Therefore, we could split the NTM strains into two subsets, one with a pattern for the 20 target compounds which clearly differed from MTB complex strains (Figures 1b and 1c compared with 1a), and another subset which had a pattern that was rather similar to the MTB complex (Figures 1d and 1e compared with 1a). In Figure 1c, the fingerprint pattern of the normalized areas of the 20 compounds is given for *M. marinum*. High levels of mycocerosate markers 13 and 14 and low levels of markers 16 and 17 were observed compared to the MTB strains; markers 19 and 20 were not detected. These features enabled correct classification of *M. marinum* as NTM by our model. Testset-1 contained two *M. kansasii* type I strains (Figures 1d and 1e). These strains showed a marker pattern similar to the MTB strains, especially for the mycocerosates (markers 13, 14 and 17–20). Compounds 17–20 are important markers for MTB since they have negative beta coefficients (see Table 3). One *M. kansasii* type I strain was correctly classified as NTM (Figure 1d) but the other strain (Figure 1e) was misclassified as MTB by our model. When the level of any of the marker compounds 17–20 is higher than a certain threshold (normalized areas of markers 17–18>10% and normalized areas of markers 19–20>3% as seen in Figure 1e), the strain is wrongly classified as MTB complex. As a result, for Testset-1, all 39 strains belonging to the MTB complex (including *M. tuberculosis*, *M. africanum, M. bovis* and *M. bovis BCG*) were correctly classified, as well as all but one (*M. kansasii*) of the 16 strains of NTM. With these strains from the Netherlands, the model had 100% sensitivity (the percentage of correctly identified MTB strains) and 94% specificity (the percentage correctly identified NTM strains). The accuracy was thus 98%.

**Figure 3:**
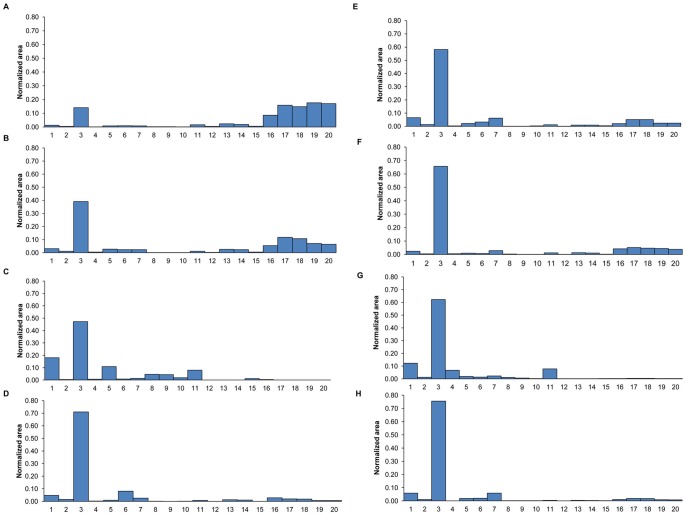
Fingerprint patterns of the normalized areas of the 20 markers in samples from South Africa. The graph shows the fingerprint patterns of the normalized areas of 20 marker compounds in different mycobacteria from South Africa with two representative *M. tuberculosis* strains (A & B), one representative NTM strain *M. intracellulare* (C), five misclassified *M. tuberculosis* strains (D–H). Note the high value of compound 3 and the low values of compound 17–20. Compounds 1–20 are identified in Table 3.

In the samples from South Africa (Testset-2), using THM-GC-MS and our 20-compound model, all 12 NTM isolates were correctly identified (see Table 5). Eighty of the 91 MTB (88%) isolates were classified correctly. The remaining 11 isolates (identified by the Bioline test and 16S rRNA sequencing as belonging to the MTB complex) were misclassified as NTM by our model. For Testset-2, the sensitivity was 88%, specificity was 100% and accuracy was 90%. The poorer performance of our model with these samples can be attributed to the very different geographical origin of the samples used for the training set and the test set. The samples from the training set came from the Netherlands whereas the samples from the test set came from South Africa. The fingerprint patterns of the normalized areas of the 20 compounds are given for some representative strains from South Africa in Figure 3. In Figures 3a and b, two different MTB strains are shown and the pattern of an NTM is shown in Figure 3c. All three were correctly classified in our model. Five of the 11 misclassified MTB isolates are shown in Figure 3d–3h. These strains were rich in compound 3 with concentrations ≥ 60%. The concentrations of the mycocerosate marker compounds 17–20 were very low. The β-coefficient values of the 20 compounds in Table 3 show that hexacosanoic acid (compound 16) and the mycocerosate markers (17–20) are important markers for the MTB complex. In the strains shown in Figure 3d-3h the low levels of these markers resulted in their misclassification as NTM strains. It is relevant to note that our model uses information about all 20 compounds. However, the most important compounds for the identification of MTB are compounds 16–20. If these are present in an MTB strain at very low levels or are below the detection limit, the model will misclassify the MTB as NTM.

**Table 5:** Results of the analysis of 103 primary isolates from patients from Stellenbosch, South Africa using THM-GC-MS and the 20-compound model.

Mycobacterial species/strain	Classification by THM-GC-MS^1^ using the 20- compound model
80 strains *M. tuberculosis*	80 strains MTB complex^2^
11 strains *M. tuberculosis*	11 NTM^3^
2 strains *M. intracellulare*	2 strains NTM
2 unknown strains Bioline test Negative	2 strains NTM
2 strains *M. peregrinum/M. septicum*	2 strains NTM
2 strains *M. avium*	2 strains NTM
1 strain *M. lentiflavum*	1 strain NTM
2 strains *M. gordonae*	2 strains NTM
1 strain *N. shimofusensis*	1 strain NTM

1 THM-GC-MS  =  Thermally-assisted hydrolysis and methylation gas chromatography-mass spectrometry.

2 MTB complex  =  *M. tuberculosis* complex.

3 NTM  =  Non-tuberculous mycobacteria.

The mycocerosate markers 13, 14, 17–20 (Table 3) have also been found by other researchers to be relevant MTB markers [19], [20]. However, we have found some of these markers are also present in some other pathogenic and opportunistic mycobacteria such as *M. kansasii, M. marinum, M. gastri, M. ulcerans* and *M. leprae.* Mycocerosates are multimethyl branched fatty acids present in phthiocerol dimycocerosates (PDIMs), diacyltrehaloses (DATs), polyacyltrehaloses (PATs) and phenolphthiocerol dimycocerosates (PGLs) [21] and are released by the THM treatment [18]. The PDIMs are highly stable waxes, composed of mixtures of long-chain mycocerosic acids esterified to the phthiocerols, long-chain C34 and C36 diols [20]. The differences in the amount of PDIMs in various *M. tuberculosis* strains and the fact that PDIMs are also present in a few strains of NTM may potentially give rise to confusion if PDIMs alone are used as the feature to distinguish MTB from NTM. Recently, O'Sullivan and coworkers used THM-GC-MS to look for mycoserates (with markers corresponding with our compounds 17–20) in sputum [22]. Their method had a sensitivity of only 61% with a specificity of 71% to detect MTB in 395 sputum samples from Zimbabwe [22]. Our own study suggests that this rather poor sensitivity may be due to the very low levels of mycocerosates found in some MTB strains. On the other hand, as noted by O'Sullivan and coworkers, the presence of high levels of matrix compounds from sputum which elute at similar retention times can easily result in false positive and false negative results, and hence a low specificity and sensitivity [22]. With sputum, overloading of the GC-MS is a potential problem and the inherent lack of robustness of GC-MS may render the approach, as it stands, unsuitable for routine use in diagnostic laboratories.

The performance of our model for the Dutch testset-1 was similar to that achieved previously when measured with the Dutch training and validation set [17]. In the present study the results for the South African samples were slightly less accurate, most likely because the training set used to establish the model lacked strains from South Africa. For optimum performance the training set used to derive the model should consist of locally occurring strains and should include an adequate number of NTMs (preferably approximately 50%). However, in our model development only 12 NTM strains were available from South Africa, which precluded us from building a more location-specific model. In a recent study Olivier and Loots found that GC-MS and multivariate statistical analysis could be used to distinguish *M. kansasii*, *M. avium*, *M. tuberculosis* and *M. bovis* from each other using 12 metabolite markers [23]. They used a modified Bligh-Dyer extraction, methylation under basic conditions, followed by hexane extraction. Three of their markers (C17, TBSA and C32 mycocerosate) were the same ones we found using THM-GC-MS. Our method has the advantage over theirs that it needs no sample treatment since suspensions of heat inactivated mycobacteria can be analyzed directly. Also, our fully-automated procedure makes the method robust and easy to perform. O'Sullivan and coworkers used a methanol/petroleum-ether extraction method [22]. We have run a petroleum ether extract of mycobacteria through the THM-GC-MS and compared the results with those obtained using our simple extraction method (data not shown). The patterns were the same, confirming that the efficiency of our approach is the same as that for petroleum ether extraction.

**Figure 4:**
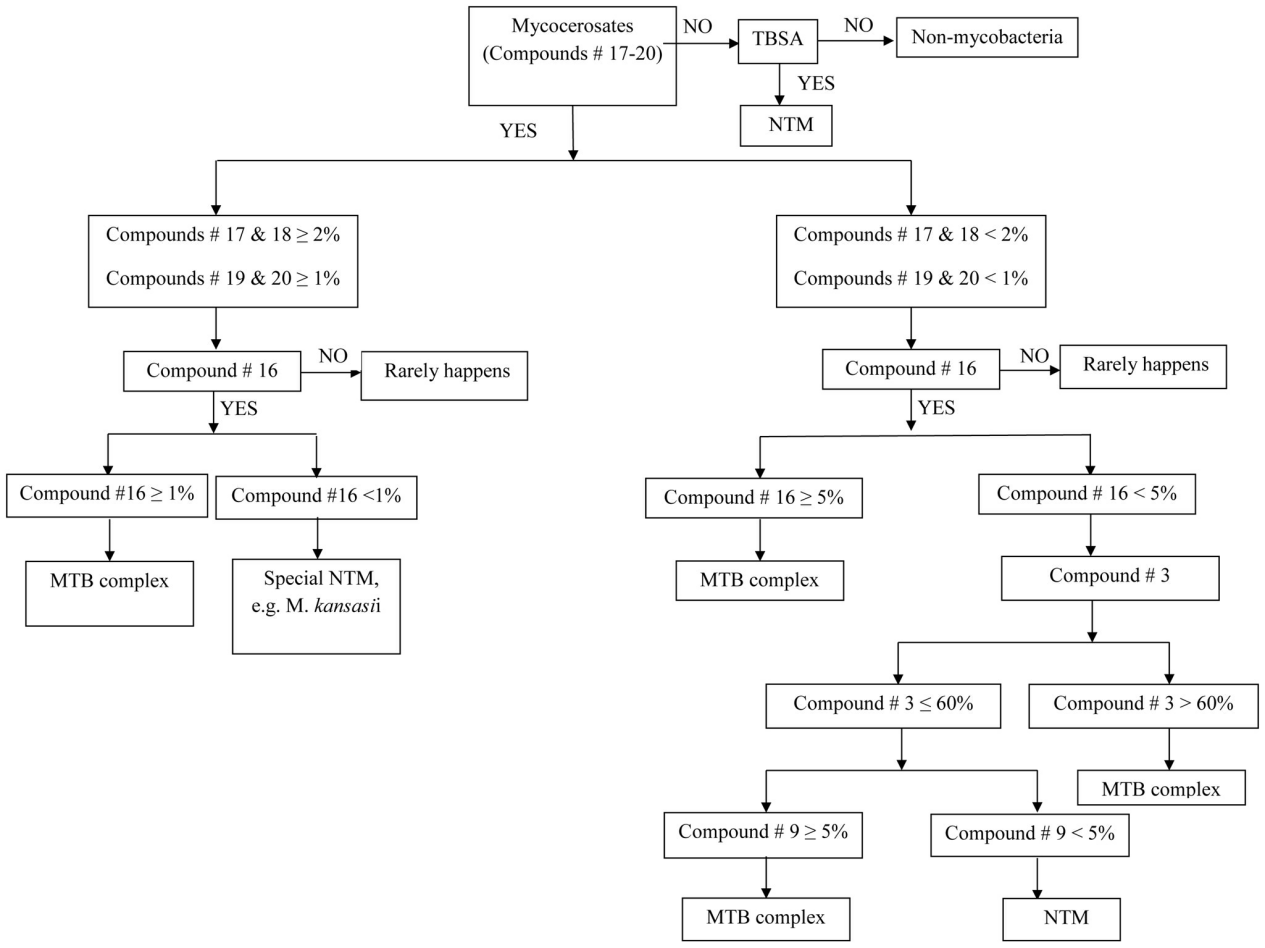
Algorithm derived using manual inspection of 103 samples from South Africa. The graph shows a decision tree for the differentiation of *M. tuberculosis* complex and non-tuberculous mycobacteria.

To improve the sensitivity of the model for South African strains whilst maintaining a high specificity, we built a decision tree using chemical intuition and experience. The method is based on normalized areas of the 20 target compounds, ranking them in order of importance with regard to distinguishing MTB and NTM strains. The tree diagram obtained this way is given in Figure 4. The algorithm uses the normalized areas of four mycocerosates (compounds 17–20), hexacosanoic acid (compound 16), tuberculostearic acid (compound 8), palmitic acid (compound 3) and the disaccharide (compound 9). The decision tree was built using all 103 samples in Testset-2 from South Africa. We applied the algorithm to the 100 samples from the Netherlands consisting of the 56 samples of Testset-1 and 44 samples from the training set 17]. The performance of the newly developed decision tree model was excellent. All the samples were correctly classified when this algorithm was used.

In addition to the visual tree model we have explored an alternative method for obtaining a decision-tree type model based on a CART (Classification And Regression Trees) method. CART is a simple but powerful method for multivariate classification and regression based on a series of subsequent binary partitions using multivariate data [24]. This method creates an algorithm from a decision-tree strategy constructed in a systematic way, rather than by intuition, as described in the previous paragraph. As different classification trees may be constructed, the “best” tree model is optimised and further “pruned” using a cross-validation strategy. To perform the optimization based on a cross-validation is crucial, since tree methods are prone to over-fitting. Also, care should be taken to avoid over-optimistic models.

**Figure 5:**
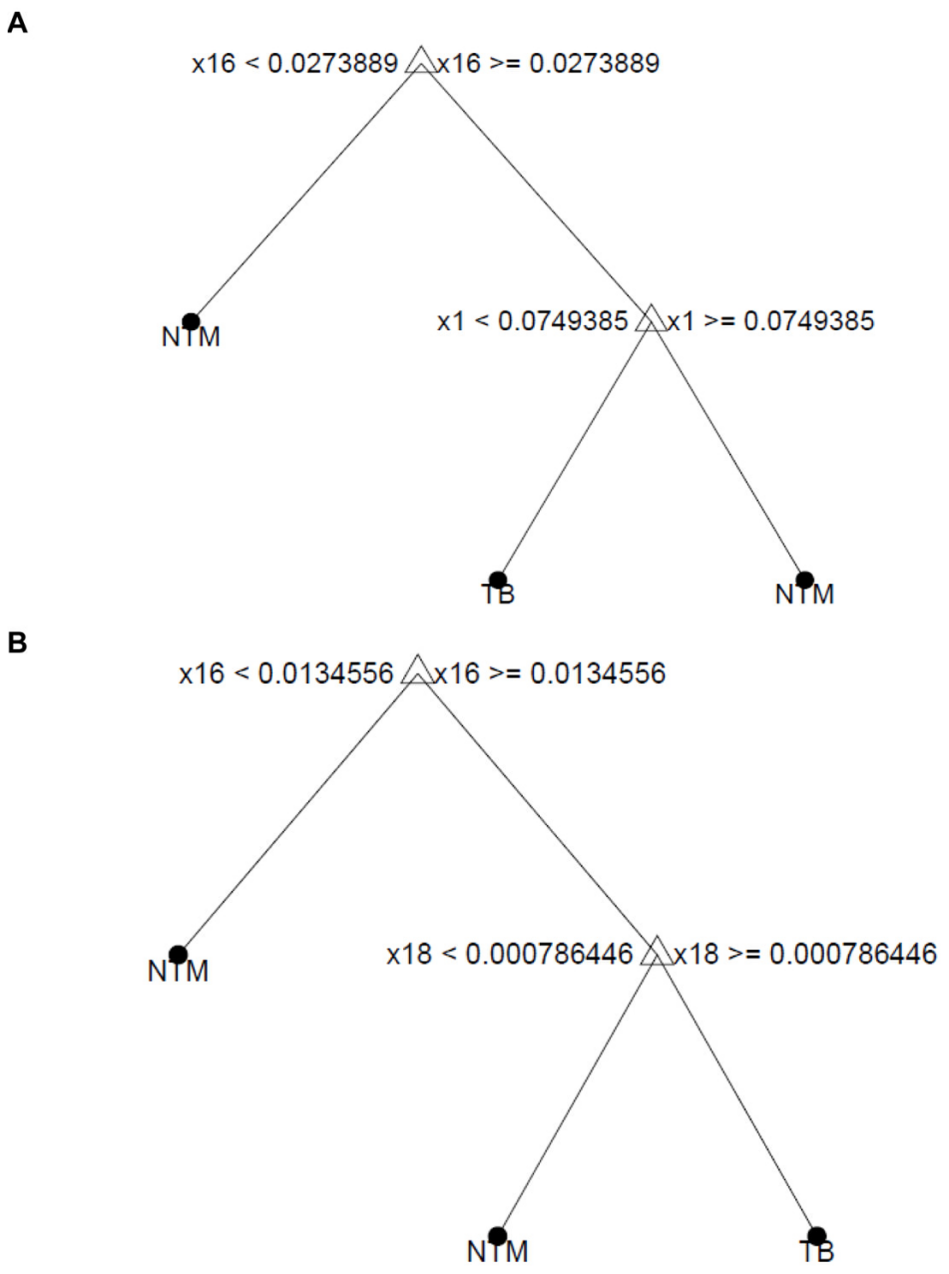
CART models using computer. (A) used 44 samples from the Netherlands as training and validation sets and 159 samples as test sets (Testset-1, 56 Dutch samples and Testset-2, 103 samples from South Africa); (B) used all 203 samples as training and validation sets.

We fitted two CART models. For the first model we considered the data set consisting of 15 MTB complex strains and 29 NTM strains from the Netherlands used in our previous study [17]. We used this data set for calibration and cross-validation. The optimised tree model (after cross-validation) is shown in Figure 5a. The model is extremely simple: it only uses two compounds, #16 and #1, which, as expected, have one of the lowest and highest β-coefficients respectively (Table 3). The model yielded an overall accuracy (cross-validated) of 95% (similar to the method based on the β-coefficients, Table 3). When applied to Testset-1 the accuracy was 96%. Indeed a good accuracy is expected, since the Testset-1 consists of samples from the same geographical region. As with our other models, when this model was applied to Testset-2, the accuracy decreased to 90%.

Figure 5b depicts the CART model using the data sets derived from all 203 samples together as training and validation sets. The model has been optimised by cross-validation, yielding an accuracy of 99.5% (cross-validated). It follows that, in agreement with the model shown in Figure 4, it is possible to construct a classification model with nearly 100% accuracy. Furthermore, as this model has been fitted using data from different geographical origins, the high degree of accuracy suggests that it is possible to create a global model which is applicable to different geographical regions. The CART model from Figure 5b makes use particularly of compounds #16 and #18 and is also very simple. Compound #16 was a crucial element in the manual decision tree proposed in Figure 4. Compounds #17–20 were also used in that model. As these compounds are normally correlated, the CART model finds it optimal to use only one of them as a classifier, confirming the usefulness of the algorithm depicted in Figure 4. Moreover, as the accuracy of the CART model was determined after cross-validation, there is more certainty that our high accuracy is not over-optimistic. The fact that the degree of accuracy obtained by cross-validation in Figure 5a is similar to the one obtained with the Testset-1 can also be considered as an indirect proof that the accuracy of the model depicted in Figure 5b is not over-optimistic.

The use of a subset of the 20 compounds (as suggested with the manual decision tree model and with the CART models) does not mean that the peak areas of the compounds not participating in those models are not relevant. This is because the tree models make use of normalized peak areas, i.e. the areas of every compound are normalized to make the sum of the 20 compound areas equal to 1. Hence, in practice, the user should still measure experimentally the peak area of the 20 compounds, in order to normalize the values correctly. In principle, it could be possible to fit a tree model using information from the two compounds participating in the model only (normalization within the two compounds). From an experimental perspective, this would be highly attractive, since then the experimental measurement of the remaining 18 compounds could be skipped. However, when the tree model was fitted in this way, its performance was significantly reduced (from 99% to 86.5%).

We plan to explore if the combination of our 20-compound model, the decision tree and CART models can be applied to identify and classify MTB and NTM strains directly in sputum. If so it would pave the way to the development of a much simpler, high-throughput test to identify MTB or NTM directly in sputum. This panel of 20 compounds offers the possibility of further developments which could result in a simple test for field use in resource-constrained countries.

## Conclusion

Conventional methods for the differentiation of MTB from NTM still suffer from the limitations of speed, sensitivity and specificity. Our fully automated THM-GC-MS approach with the 20-compound model is a promising tool to differentiate MTB and NTM. Excellent results can be obtained when the training and validation sets originate from the same geographical settings. If the sample set is obtained from a different area the results may not be as good, although our method still achieved a sensitivity of 88% and specificity of 100%. We have derived two types of tree models to solve this problem. One algorithm was constructed using manual inspection of the data, which enabled correct classification of all 103 samples from South Africa and 100 samples from the Netherlands, i.e. 100% sensitivity and specificity. Another tree was fitted using a CART model. This last model was extremely simple (only two compounds included), yet highly accurate (99.5% accuracy) when the accuracy was tested using cross-validation. Although promising, these findings were derived from strains from The Netherlands and South Africa only. Future studies using strains from different areas are needed to corroborate these findings. Our final goal is to develop a micro GC and portable detector to detect and differentiate MTB and NTM directly in sputum.
